# Vitamin D_3_ Bioaccessibility from Supplements and Foods—Gastric pH Effect Using a Static In Vitro Gastrointestinal Model

**DOI:** 10.3390/molecules29051153

**Published:** 2024-03-05

**Authors:** Evangelia Pasidi, Patroklos Vareltzis

**Affiliations:** Department of Chemical Engineering, Faculty of Engineering, Aristotle University of Thessaloniki, 54124 Thessaloniki, Greece; egpasidi@cheng.auth.gr

**Keywords:** vitamin D_3_ bioaccessibility, in vitro digestion, gastric pH

## Abstract

Vitamin D_3_ deficiency is a global phenomenon, which can be managed with supplementation and food fortification. However, vitamin D_3_ bioaccessibility may depend on factors such as matrix composition and interactions throughout the gastrointestinal (GI) tract. This research focused on the effect of different matrices on vitamin D_3_ content during digestion, as well as the effect of pH on its bioaccessibility. The INFOGEST protocol was employed to simulate digestion. Three different types of commercial supplements, two foods naturally rich in vitamin D_3_, and three fortified foods were investigated. High-Performance Liquid Chromatography was used to determine the initial vitamin D_3_ content in the supplements and foods, as well as after each digestion stage. The results indicate that the foods exhibited higher bioaccessibility indices compared to the supplements and a higher percentage retention at the end of the gastric phase. The pH study revealed a positive correlation between an increased gastric pH and the corresponding content of vitamin D_3_. Interestingly, exposing the matrix to a low pH during the gastric phase resulted in an increased intestinal content of D_3_. Vitamin D_3_ is more bioaccessible from foods than supplements, and its bioaccessibility is susceptible to changes in gastric pH. Fasting conditions (i.e., gastric pH = 1) enhance the vitamin’s bioaccessibility.

## 1. Introduction

**Scheme 1:**
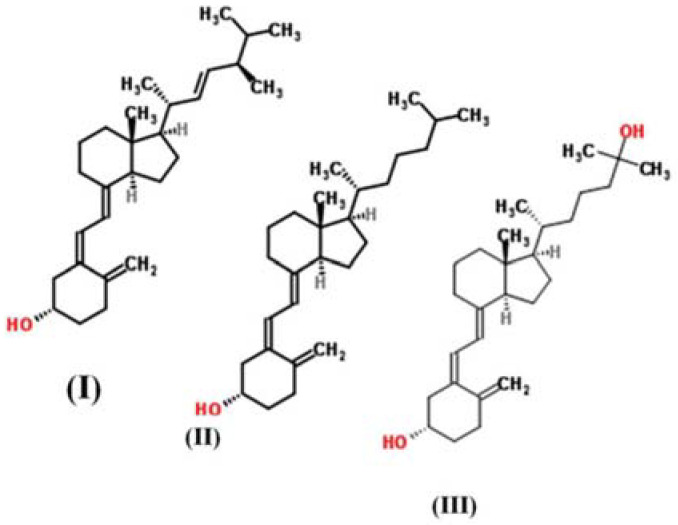
Chemical structures of main dietary forms of vitamin D: (**I**) ergocalciferol (vitamin D_2_), (**II**) cholecalciferol (vitamin D_3_), (**III**) 25-hydroxy-cholecalciferol (25(OH)D_3_) [8].

Vitamin D is a biologically active compound found mainly in the forms of ergocalciferol (vitamin D_2_), cholecalciferol (vitamin D_3_), and 25-hydroxycholecalciferol (25(OH)D_3_) (Scheme 1). Vitamin D_3_ is a micronutrient, essential for maintaining the overall health and wellness of humans, as it is associated with bone health and immune system boosting [1]. It plays a key role in calcium absorption [2] and has been proposed to act against cancer cell growth [3]. In addition, vitamin D_3_ has been linked with a lower risk of developing multiple sclerosis and rheumatoid arthritis, as well as type 1 and type 2 diabetes mellitus [4]. The protective effect of vitamin D_3_ against SARS-CoV-2 has also been examined and showed that the vitamin can potentially prevent severe illness [5]. Vitamin D_3_ can be photosynthesised through skin exposure to ultraviolet radiation [6] or ingested through foods and supplements. However, vitamin D_3_ deficiency is a global concern [7]. Supplementation, as well as the consumption of foods rich in vitamin D_3_, can aid in coping with this phenomenon. 

Supplements are available in different forms, such as tablets, capsules, or oil-emulsified drops [9]. Vitamin D_3_ in supplements may be encapsulated in microcapsules, micelles, or liposomes to increase its bioavailability [10,11,12]. A meta-analysis of several clinical studies concluded that vitamin D_3_ bioavailability is better in oil vehicles (capsules or liquid) than in powder tablets (cellulose or lactose) or ethanol [13]. However, another study testing oil and tablets showed that they were equally efficient in raising serum 25-hydroxyvitamin D, though the authors speculated that these results may be due to the timing of measuring serum concentrations [14]. 

Foods can either naturally contain vitamin D_3_, such as fish and eggs, or be fortified with the vitamin, such as milk, orange juice, plant oils, flour, bread, and cereals. Different food matrices can result in different bioaccessibility and bioavailability levels of the vitamin. The structure of the food matrix, the amount and type of dietary lipids (chain length and degree of saturation), and the dietary fibres can affect the final bioavailability [8,15]. Foods fortified with vitamin D_3_ have demonstrated comparable effectiveness to supplements in increasing serum 25(OH)D_3_ levels [16,17]. It has also been suggested that vitamin D_3_ absorption is protein-mediated at low concentrations, such as that found in dietary sources, while at high pharmacological concentrations, the absorption mechanism shifts to passive diffusion [18]. The differences observed between different foods and supplements indicate that research on various matrices is necessary. 

When a vitamin D_3_-containing matrix is ingested, it undergoes physiological conditions encountered during digestion, including enzyme activity and pH fluctuations. The digestive process is initiated in the mouth with amylase catalysing starch hydrolysis, followed by the stomach, where proteins and lipids are hydrolysed by pepsin and gastric lipase, forming gastric chyme [19]. As the gastric chyme moves to the intestine, pancreatin further breaks down the food with assistance from intestinal peristaltic movements. Pepsin and trypsin may play a role in releasing vitamin D_3_ from its food matrix by disrupting the binding of proteins to vitamin D_3_. Digestive enzymes in the duodenum, including amylase, lipase, and protease, continue to liberate vitamin D_3_ from its food matrix [15]. The released vitamin D_3_ integrates into the mixed micelles formed during digestion, consisting of phospholipids, cholesterol, lipid digestion products, and bile salts [8,15,20]. The composition of mixed micelles is influenced by the types of lipids present during digestion [21,22]. 

pH variation is another critical factor that might impact the final bioaccessibility of vitamin D_3_. A lower pH has been shown to lead to a decreasing stability of vitamin D_3_ [23]. Vitamin D_3_ is isomerised to isotachysterol at acidic pH [24]. Encapsulation of the vitamin has been proposed to protect it from degradation at different pH values [25]. Many encapsulation techniques and materials, such as β-lactoglobulin [26], ovalbumin–pectin nanocomplexes [27], gum arabic, maltodextrin, whey protein concentrate, and soy isolate protein [28], have been used to produce systems that are stable under different pH conditions [25]. Food intake alters the basal gastric pH. Different food compositions result in varying gastric pH values, which may take up to 3 h to return to basal levels [29]. Consequently, supplements taken after different foods or during fasting may encounter different gastric pH conditions. The timing of supplement intake following food consumption can also lead to variations in the encountered pH values [30].

The aim of this research was to investigate the influence of diverse matrices (including natural foods with vitamin D_3_ with or without heat treatment, fortified foods, and supplements) on the fate of vitamin D_3_ at different stages along the gastrointestinal (GI) tract. Using the INFOGEST protocol, these matrices underwent in vitro digestion, and the vitamin content was determined at different stages of the protocol to evaluate its bioaccessibility. Additionally, this study investigated the effect of gastric pH by testing four different pH values to simulate conditions during fasting and the consumption of various foods. The findings from this research contribute to a deeper understanding of how each digestion stage influences vitamin D_3_ and the impact of gastric pH variations on its bioaccessibility.

## 2. Results

Foods naturally containing vitamin D_3_ (eggs and salmon), fortified foods (milk, cereals, and sour cherry juice), and supplements (tablets, capsules containing an oil-based emulsion, and oil-based liquid drops) were subjected to in vitro digestion (INFOGEST protocol) [31]. High-Performance Liquid Chromatography (HPLC) was used to determine the vitamin D_3_ content and losses at each stage. The eggs and salmon were thermally processed until their core temperature reached 70 °C [32] before being subjected to digestion. The effect of gastric pH variation was also examined by subjecting the vitamin D_3_ liquid oil-based supplement to four different gastric pH values. 

### 2.1. Vitamin D_3_ Content of Foods and Supplements

**Figure 1:**
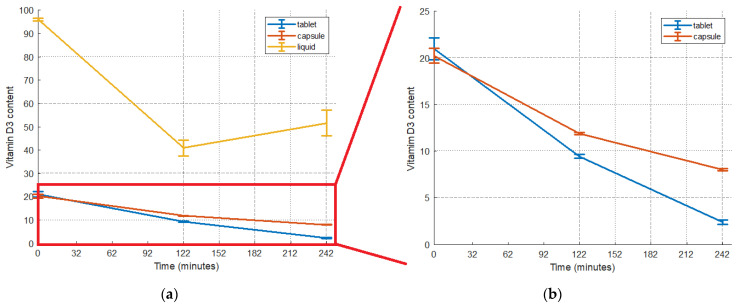
Detected vitamin D_3_ content during in vitro digestion of (**a**) commercial supplements: tablet; capsule; liquid. (**b**) Close view of tablet and capsule (tablet in μg/tablet, capsule in μg/capsule, and liquid in μg/mL). On *x*-axis, 0, 122, and 242 minutes refer respectively to the initial content, content after gastric stage, and content after intestinal stage.

The detected content of the vitamin in the tablet, capsule, and liquid supplement was 20.99 ± 1.17 μg/tablet, 20.24 ± 0.78 μg/capsule, and 95.93 ± 0.64 μg/mL, respectively (Figure 1, t = 0 min). The liquid supplement had the highest content, followed by the tablet and capsule, which had similar contents. 

The fortified foods had a higher vitamin content than the natural foods, as expected (Table 1). Between the two natural foods examined, the salmon had a higher vitamin D_3_ content than the eggs, as seen in other studies [33]. An HPLC analysis of the egg and salmon showed a second peak, before vitamin D_3′_s peak (Figures S3 and S4—Supplementary file), which may correspond to the hydroxylated form 25(OH)D_3_ [34]. This form is naturally present in these foods [35,36].

### 2.2. Vitamin D_3_ Bioaccessibility

#### 2.2.1. Vitamin D_3_ Bioaccessibility from Supplements

**Figure 2:**
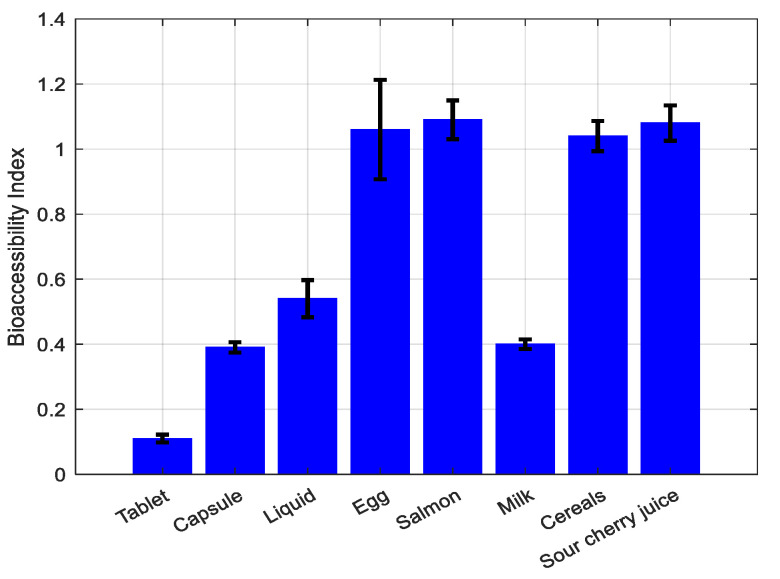
Bioaccessibility indices of supplements: tablet; capsule; liquid; and foods: egg, salmon, milk, cereals, sour cherry juice.

The bioaccessibility index (BI) shows the amount of vitamin D_3_ remaining after digestion processes and available for absorption, and it was calculated according to Equation (2). In Figure 1, the remaining detected content of vitamin D_3_ is presented. Among the supplements, the liquid one had the highest bioaccessibility, followed by the capsule and the tablet (Figure 2).

At the end of the gastric phase, the vitamin D_3_ content in the tablet, capsule, and liquid supplements was reduced by 55%, 41%, and 43%, respectively. Further losses at the end of the intestinal phase were recorded (75% and 20% for the tablet and capsule). On the contrary, in the case of the liquid supplement, there appeared to be a 25% increase in the vitamin D_3_ content in the intestinal stage compared to the gastric stage. Greater losses were observed for the tablet at each stage. The reduction in the vitamin content during the intestinal phase was more significant for the tablet compared to that during the gastric phase. In contrast, for the capsule, the reduction was more pronounced for the gastric content compared to the initial content. 

#### 2.2.2. Vitamin D_3_ Bioaccessibility from Foods

The detected vitamin contents in each digestion step of the foods, as well as the corresponding bioaccessibility, are presented in Table 1. The sour cherry juice, egg, salmon, and cereals had BIs around 1. The milk had the lowest BI at 0.40, which is rather low in comparison with the other samples. The foods, except for the milk, exhibited higher BIs than the supplements, as shown in Figure 2.

The thermal processing of the egg and salmon seemed to decrease the vitamin D_3_ content by 43% and 25%, respectively (Table 1). 

In the natural food samples, the vitamin D_3_ content seemed to increase after the gastric step. The vitamin D_3_ content in the gastric chyme of the egg and salmon samples was increased by 33% and 48%, respectively, compared to the initial concentration. The intestinal content compared to the gastric content was decreased by 29% for the eggs and by 26% for the salmon. 

The results for the fortified food samples showed a 60% and 5% decrease in the vitamin content in the gastric phase for the milk and cereals, respectively. For the sour cherry juice, there was a slight increase (4%) in the gastric content compared to the initial content. For the milk samples, there was no significant difference between the gastric and intestinal contents. The cereals and sour cherry juice showed an increase in the intestinal content (10% and 3%, respectively).

The gastric step seemed to have a greater impact on vitamin D_3_ for all food samples, either by increasing or decreasing the content. 

### 2.3. Gastric pH Effect on Vitamin D_3_ Bioaccessibility

Four different pH values were simulated to investigate the effect of the gastric stage pH on vitamin D_3_ bioaccessibility. The sample tested was the liquid supplement, as it was the most bioaccessible among the supplements. The gastric and intestinal contents of the vitamin, as well as the calculated BIs, are presented in Table 2.

There was a profound effect of the gastric digestion step on the vitamin content. The vitamin’s decrease during this stage ranged from 44 to 58%. D_3′_s gastric content was the highest at pH 7 and the lowest at pH 1 (*p* < 0.05). Even at pH 7, there was a 44% decrease in the vitamin D_3_ content. This suggests that vitamin D_3_ stability might be affected not only by pH but also by the presence of other components of gastric fluids. A low pH has been shown to negatively affect vitamin D_3_ [23]. Different pH values may have caused the degradation of vitamin D_3_ to isomers [34,37].

At every pH level, there was an increase in the vitamin D_3_ content at the end of the intestinal digestion phase, except for at pH 7. The percentage increases were 78%, 26%, and 10% at pH 1, 3, and 5, respectively. The lower the pH of the gastric phase, the higher the increase in the vitamin D_3_ content in the intestinal phase. On the contrary, when the sample was exposed to gastric pH 7, a notable 23% reduction in the vitamin D_3_ content was observed, from 54 μg/mL after the stomach phase to 41 μg/mL after the intestinal phase. Exposure to the lowest pH value of 1 resulted in the highest BI, while pH 7 led to the lowest BI. pH values 3 and 5 had similar BIs.

**Figure 3:**
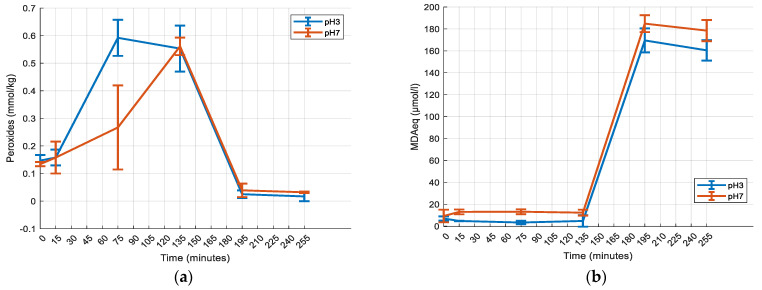
Effect of gastric pH on (**a**) primary oxidation and (**b**) secondary oxidation of sunflower seed oil. On *x*-axis, 0 min refers to the initial concentration, 15 min refers to the concentration after oral phase, 75 and 135 min refer to the concentration after the first and second hours of gastric phase, and 195 and 225 min refer to the concentration after the first and second hours of intestinal phase.

To determine the possible effect of the carrier’s oxidation (sunflower oil) on the vitamin D_3_ content in each digestion stage, sunflower seed oil oxidation was investigated at two different pH values (Figure 3). Primary oxidation was more profound at pH 3 than at pH 7. The concentration of primary oxidation products peaked during the gastric phase at 75 min at pH 3 and at 135 min at pH 7. Even though oxidation at pH 7 was significantly delayed during the gastric phase, it reached the same peak concentrations of oxidation products at pH 3 (*p* ≥ 0.05). Secondary oxidation peaked during the intestinal phase of digestion, when primary oxidation products had the lowest concentrations (195 min). The concentration of secondary oxidation products was greater at pH 7; however, the difference was not statistically significant (*p* ≥ 0.05).

## 3. Discussion

Supplements and foods containing vitamin D_3_, either naturally or from fortification, can be used to battle vitamin D_3_ deficiency [38,39]. When ingested, vitamin D_3_ is exposed to GI tract conditions, which can affect the stability of the vitamin and its final bioaccessibility.

Of the three commercial supplements, the oil-based liquid drops had the highest vitamin content. The in vitro digestion of the supplements showed the highest BI for the oil-based liquid drops, followed by the capsule and the tablet, which is in accordance with previous studies testing vitamin D_3_ bioavailability [13]. Vitamin D_3_ is a lipophilic vitamin and is more stable in oil vehicles [23]. 

The tablets exhibited higher gastric losses compared to the capsules and liquid supplements, with the intestinal stage exerting a more significant impact on the vitamin content of the tablets. Conversely, for the capsules, the gastric stage had a more pronounced effect. In the case of the liquid supplement, there was a decrease in content from the initial to the gastric stage, followed by an increase from the gastric to the intestinal stage. This phenomenon may be attributed to the enhanced release of the vitamin from its matrix during this stage, potentially facilitated by the action of pancreatin on the oil matrix (sunflower oil). A similar behaviour was noted for carotenoids, as they were undetected in the gastric stage but present in measurable concentrations during the intestinal stage. The authors attributed this outcome partly to the presence of pancreatin in the intestinal stage [40]. Additionally, the antioxidant capacity of the α-tocopherol present as an additive in the liquid supplement may have protected vitamin D_3_ from degradation during in vitro digestion [41]. Differences in the initial concentrations among the supplements might also have contributed to the different behaviours during digestion. Previous research has shown that the BI of omega-3 supplements can be dependent on the initial concentration of the lipophilic components [42].

Heat treatment can adversely affect the vitamin D_3_ content of foods by decreasing it, depending on the method of heating [43,44]. In our study, the thermal processing of egg and salmon decreased the vitamin D_3_ content, with the egg being more affected than the salmon (42% vs. 25% decrease, respectively). Vitamin D_3_, as a lipophilic vitamin, may be better protected in salmon than eggs, as salmon has a greater lipid content. This can result in a greater retention of vitamin D_3_ in salmon after thermal processing. Vitamin D_3_ converts to pre-vitamin D_3_ reversibly when heated, especially at higher temperatures [37]. The reversibility of this conversion may be the explanation for the increase observed in the gastric step of both the eggs and salmon after the heat treatment.

The eggs and salmon had a lower vitamin D_3_ content than the fortified foods, as expected. The salmon had a higher content than the eggs, as shown in other studies [33]. From the fortified foods, the milk had the highest content, followed by the cereals and sour cherry juice. The foods exhibited higher BIs than the supplements, apart from the milk, which had a rather low BI, closer to that of the supplements. Previous research has shown that naturally formulated vitamin D_3_ extracted from agricultural products had a higher bioaccessibility than synthetic vitamin D_3_ [45]. An investigation on vitamin E bioaccessibility revealed that the incorporation of vitamin E-loaded Pickering emulsions into foods led to an increased bioaccessibility of the vitamin, surpassing the bioaccessibility observed when the emulsion was digested alone. This observation was attributed to the natural presence of macronutrients in foods [46]. These findings are in accordance with our results concerning the better bioaccessibility of vitamin D_3_ from foods. 

In the natural foods, the gastric phase showed a beneficial impact, leading to an increase in the vitamin D_3_ content, while the intestinal phase adversely affected the vitamin’s content. The observed increase during the gastric step may be due to the release of the vitamin from the food matrix, which made it available for detection. The percentage increase in the gastric step was higher than the percentage decrease in the intestinal step, which indicates that the gastric step had a greater effect on the vitamin D_3_ content. The intrinsic antioxidant mechanisms of fish tissue may have acted as a protective agent for vitamin D_3_. Greater lipid oxidation may cause the degradation of the vitamin [47,48]. The enzymatic antioxidants in fish, such as glutathione peroxidase (GPx), can reduce lipid peroxides [49], thus protecting vitamin D_3_ by decreasing lipid oxidation. Vitamin C and vitamin E, which act as antioxidants, are also present in fish tissue [50]. These vitamins may also have functioned as protective agents against vitamin D_3_ degradation. Regarding eggs, their digestion causes the release of amino acids and antioxidant peptides, which raise their antioxidative capacity while preserving the bioaccessibility of their naturally occurring antioxidants, zeaxanthin and lutein [51,52,53]. This phenomenon may have aided in protecting the vitamin D_3_ present in the eggs during digestion.

Among the fortified foods, the milk exhibited a notable reduction in the vitamin D_3_ content from the initial to the gastric step. However, the decrease from the gastric to the intestinal step was comparatively minimal and lacked statistical significance. This suggests that, like the natural foods, the gastric step had a more pronounced impact on the milk. Previous studies have shown a low bioaccessibility of vitamin D_3_ in milk [54,55]. The bioaccessibility of vitamin D_3_ in milk has been found to vary in different types of milk (skim, partially defatted, whole, and infant formula milk) [54], indicating the possible role of not only the fat content but also the type of fats present in the matrix. The low bioaccessibility may also be attributed to the interference of calcium with vitamin D_3_ absorption. Previous research on fortified plant-based milk has shown that calcium forms insoluble calcium soaps that trap the vitamin [56]. Similar results were obtained for water-in-oil-in-water emulsions, where vitamin D_3_ bioaccessibility was reduced in the presence of calcium [57]. Furthermore, vitamin D_3_ can bind to milk proteins, such as β-lactoglobulin and β-casein, under both acidic and alkaline conditions with different binding affinities [58]. This may also have resulted in decreased bioaccessibility, as vitamin D_3_ may not be able to be separated from milk proteins during saponification and extraction. 

The vitamin D_3_ content in the cereals decreased in the gastric stage compared to the initial content, while for the sour cherry juice, a slight increase after the gastric stage was observed. After the intestinal stage, there was an increase in the content compared to the one in the gastric stage for both foods. A study on vitamin D_3_ bioaccessibility from test meals showed that semolina meal had the highest bioaccessibility [59], though not as high as in our study. The cereals used in this study contained whole wheat flour and corn semolina, which are high in antioxidants [60,61]. A study on the in vitro digestion of juice extracts found that the content of some phenolic acids and flavonoids increased either during the gastric stage or the intestinal stage, as well as that of some monosaccharides and oligosaccharides, which was attributed to the increased release during digestion [62]. The antioxidant capacity of these compounds during digestion was maintained at elevated levels. Antioxidants have been shown to protect vitamin D_3_ against degradation [41,63]. The behaviour of vitamin D_3_ during the cereal and juice digestion can be attributed to its increased release during digestion, as well as the antioxidant capacity of the phenolic acids and flavonoids present in the cereals and juice, which may have acted as protective agents against vitamin D_3_ degradation.

Regarding the effect of pH on the vitamin D_3_ liquid supplement, there were two main observations: On the one hand, in the gastric phase, the lower the pH, the higher the decrease in D_3_. On the other hand, exposure to a lower pH during the gastric phase led to a higher content of vitamin D_3_ in the intestinal phase; i.e., the content of vitamin D_3_ was higher when the matrix was exposed to pH 1 and lower when exposed to pH 7. A study on vitamin D_3_ stability in aqueous solutions found that a lower pH had a negative effect on its stability [23]_._ The stability and content of vitamin D_3_ in the GI tract may be affected by lipid oxidation, hydrolysis, and enzyme action. Metal ions, present in the gastric chyme, can also destabilise vitamin D_3_, as its degradation may be catalysed by them [23]. In this case, the matrix of the supplement consists of sunflower seed oil, which is not affected by the pepsin present in the gastric phase, as pepsin is a proteolytic enzyme [64]. The decreased content at low pH values can also be attributed to the faster primary oxidation of sunflower seed oil at lower pH values, as lipid oxidation can affect vitamin D_3_ by promoting its degradation [47,48]. The intestinal content is affected more by gastric pH changes. A lower pH leads to greater lipid hydrolysis and the release of free fatty acids, which are mixed micelles’ structural components [20]. More free fatty acids can form more mixed micelles available to incorporate vitamin D_3_, which may lead to better bioaccessibility. A study found that sunflower oil hydrophilicity increases as the pH decreases [65]. Decreased hydrophobicity may affect the formation of mixed micelles regarding their size, shape, and stability, which, by extension, can affect the vitamin’s bioaccessibility. The increased content of vitamin D_3_ in the intestine could also be attributed to the isomerisation processes that take place at different pH values. Vitamin D_3_ is isomerised to isotachysterol under acidic conditions [37], as well as lumisterol and tachysterol [34]. The isomerisation to tachysterol and lumisterol can be reversed, and pre-vitamin D_3_ is formed [66], which is then converted to vitamin D_3_. The lower pH in the gastric stage may have caused the vitamin’s isomerisation (Figure S5—Supplementary File). Based on the elution order of vitamin D_3_ and its isomers from similar published HPLC analysis results, it is suggested that the three peaks in Figure S5 (A, B, and C—Supplementary File) may correspond to isotachysterol, lumisterol, and pre-vitamin D_3_ [34,37]. As the gastric pH increases, the isomerisation processes can be of a smaller magnitude. This phenomenon, in combination with lipid oxidation, may explain the decrease in the D_3_ content in the gastric phase, as well as the corresponding increase observed in the intestinal stage. However, it is important to exercise caution when interpreting these findings, as vitamin D_3_ is prone to isomerisation and degradation under diverse conditions. This makes its stability in food products potentially uncertain and its analysis challenging. Early studies suggest that factors like the substrate/reactant ratio, solvents, and time can have varying impacts on the generation pathway of vitamin D isomers [44].

This research highlights that vitamin D_3_ is more bioaccessible from foods than supplements, and its bioaccessibility is susceptible to changes in gastric pH. Even though exposure to low gastric pH values, i.e., pH = 1, led to a lower detected vitamin D_3_ content, the corresponding intestinal content significantly increased. The mechanism(s) behind this phenomenon should be further explored. It is crucial to understand the behaviour and stability of vitamin D_3_ during digestion, as its effectiveness when consumed through foods or supplements relies on its bioaccessibility. Understanding how vitamin D_3_ interacts with other components in the digestive system and under GI conditions is essential for developing supplements and foods that optimise its stability and absorption. 

## 4. Materials and Methods

### 4.1. Chemicals and Reagents

Bile bovine dried, potassium chloride (KCl), calcium chloride (CaCl_2_(H_2_O)_2_), and magnesium chloride (MgCl_2_(H_2_O)_6_) were purchased from Merck & Co. (Rahway, NJ, USA). Sodium chloride (NaCl), sodium sulphate (Na_2_SO_4_), potassium dihydrogen phosphate (KH_2_PO_4_), potassium hydroxide (KOH), hydrochloric acid 37% (HCl), methanol (CH_3_OH), and ethanol (C_2_H_5_OH) were purchased from Chem-Lab NV (Zedelgem, Belgium). Diastase (α-amylase, malt diastase), porcine pepsin, pancreatin, and ammonium carbonate (NH_4_)_2_CO_3_ were purchased from Central Drug House Ltd. (New Delhi, India). Sodium hydroxide (NaOH) was purchased from Lach-Ner Ltd. (Neratovice, Czech Republic). Ascorbic acid (vitamin C, C_6_H_8_O_6_) was purchased from Sigma-Aldrich (Buchs, Switzerland). Hexane (H_3_C(CH_2_)_4_CH_3_) was purchased from Avantor Performance Materials (Radnor, PA, USA). Vitamin D_3_ standard was purchased from Carl Roth GmbH + Co. KG (Karlsruhe, Germany). All the chemicals and reagents used in this study were of analytical or HPLC-grade. The food samples tested were purchased from local vendors, while supplements were purchased from local pharmacies.

### 4.2. Digestion Procedure

Digestion was simulated in vitro using the INFOGEST protocol [31]. Enzyme activity must be determined for each enzyme used. In this study, amylase (mouth), pepsin (stomach), and pancreatin (intestine), as well as bile bovine, were used. The activity of enzymes not declared by the manufacturer was calculated according to the protocol. Simulated digestion fluids were prepared according to the protocol, containing KCl, KH_2_PO_4_, NaCl, MgCl_2_(H_2_O)_6_, (NH_4_)_2_CO_3_, HCl, and Cacl_2_(H_2_O)_2_. CaCl_2_(H_2_O)_2_ was added immediately before use at each step due to precipitation issues.

The samples used for the digestion experiments were 3 different types of supplements (tablets, capsules containing an oil-based emulsion, oil-based liquid drops), naturally containing vitamin D_3_ foods (eggs, salmon), and fortified foods (milk, cereals, sour cherry juice).

For each food, 5 g was used in the first step. For the tablet and capsule, an amount corresponding to 1200 IU was used, while for the liquid supplement, 5000 IU was used (diluted with water to 2 mL final volume). Solid foods were diluted and minced to achieve a paste-like consistency. Thermal processing (70 °C core temperature for 15 s) of eggs and salmon was conducted by heating the samples in a water bath [32]. Gastric pH effect experiments were conducted using the supplement with the highest bioaccessibility, as determined from the first round of experiments.

#### 4.2.1. Oral Phase

Firstly, the amount of sample was weighted, and simulated salivary fluid (SSF) 1.25× was added. Distilled water was added to reach 1× concentration of SSF. If the sample contained starch, amylase (75 U/mL) was also added. The sample was stirred for 2 min at 37 °C (ONE 14-SV 1422, Memmert, Schwabach, Germany).

#### 4.2.2. Gastric Phase

Simulated gastric fluid (SGF) 1.25×, pepsin (2000 U/mL), and distilled water were added to the mixture at the end of the oral phase. pH adjustment to 3 (protocol value), 1, 4, or 7 (for the pH study) was achieved by adding HCl 1 M (pH 211, HANNA instruments, Woonsocket, RI, USA). The sample was gently shaken for 2 h at 37 °C (ONE 14-SV 1422, Memmert, Germany).

#### 4.2.3. Intestinal Phase

Simulated intestinal fluid (SIF) 1.25× and bile salts (10 mM) were added to the gastric chyme. The mixture was stirred for 30 min at 37 °C until complete bile solubilisation. Afterwards, pancreatin (100 U/mL trypsin activity) was added, the pH was adjusted to 7 (NaOH 1 M) (pH 211, HANNA instruments, USA), and distilled water was added. The sample was stirred for 2 h at 37 °C (ONE 14-SV 1422, Memmert, Germany).

All samples were stored at −20 °C until further analysis. 

### 4.3. Vitamin D_3_ Isolation

#### 4.3.1. Samples with Saponification

The isolation method used was based on Yanhai et al. [67], with some modifications. Raw and thermally processed food were diluted in an appropriate amount of water. The samples from the digestion steps were not diluted. To each sample, 15 g/L solution of vitamin C in ethanol in a 1:2 ratio (*v*/*v*) and 1.25 g/mL solution of KOH in water in a 2:1 ratio (*v*/*v*) were added. The sample was heated at 60 °C for 45 min with continuous stirring to achieve lipid saponification. Afterwards, the sample was cooled at room temperature and underwent 2 subsequent extractions with hexane in a 1:2 ratio (*v*/*v*). For each extraction, hexane was added to the sample and vortexed for 5 min. Then, the mixture was placed in a separating funnel until complete phase separation. The water phase was removed. The hexane phases from the two extraction steps were collected and combined. Na_2_SO_4_ was added to remove any residual water. To remove Na_2_SO_4_, the sample was filtered through filter paper (retention 10–15 μm). Subsequently, the sample was placed in a rotary evaporator at 40 °C (Laborota 4003, Heidolph, Schwabach, Germany) and evaporated to dryness. Solids were redissolved with 2 mL methanol and filtered through a 0.22 μm filter (PTFE).

#### 4.3.2. Samples without Saponification

For the supplements, the isolation of vitamin D_3_ was conducted as follows: The capsule and tablet were diluted with 5 mL of water. The liquid supplement was used undiluted. Methanol was added to the samples in a 1:2 (*v*/*v*) ratio, vortexed for 2–3 min, and placed in an ultrasonic bath (LBS1 10Lt, FALC instruments, Treviglio, Italy) for 10 min. Then, the mixture was vortexed again for 2–3 min and centrifuged at 2.938× *g* (unicen 21, Ortoalresa, Madrid, Spain) for 15 min to achieve complete phase separation. The organic phase was collected and evaporated to dryness (40 °C, Laborota 4003, Heidolph, Germany). Solids were redissolved in 2 mL methanol and filtered through a 0.22 μm filter (PTFE). 

The juice and the digestion fractions of the juice and supplements were extracted twice with hexane. The procedure followed was as described in the previous Section 4.3.1.

### 4.4. High-Performance Liquid Chromatography (HPLC)

The vitamin D_3_ concentration was determined using HPLC with a UV detector (KNAUER 1200 system, Burladingen, Germany). The column used for separation was Eurospher II 100-5 C18A (250 × 4 mm). The mobile phase was HPLC-grade methanol and 0.1% formic acid with a constant flow rate at 1 mL/min and 25 °C. The injection volume was 20 μL. The UV detector was set to a 265 nm wavelength. Vitamin D was eluted at 4.8–4.9 min.

The vitamin D_3_ concentration in each sample was determined based on a standard curve. The standard curve was constructed using the vitamin D_3_ standard (Figures S1 and S2—Supplementary File). Different concentrations of the standard in the range of 5 to 30 ppm were analysed in the HPLC system to determine the corresponding peak areas. The limit of detection (LOD) was 0.05 ppm, and the limit of quantification (LOQ) was 0.17 ppm (Tables S1 and S2—Supplementary File). 

Recovery was determined by spiking raw foods and supplements with a known amount of the vitamin D_3_ standard and analysing the sample. Recovery was calculated according to the following formula:

(1) $$\%recovery=[(A_{spiked}-A_{unspiked})/A_{standard}]\times 100\%$$

where $A_{spiked}$ is the peak area of the spiked sample; $A_{uspiked}$ is the peak area of the unspiked sample; and $A_{standard}$ is the peak area of the vitamin D_3_ standard, which was used for spiking the sample.

All values in the tables and figures were corrected based on the recovery of each sample.

### 4.5. Bioaccessibility Index

The vitamin D_3_ bioaccessibility index (BI) was calculated using the following formula:

(2) $$BI=C_{b}/C_{a}$$

where $C_{a}$ and $C_{b}$ are the amounts of vitamin D_3_ before and after digestion [68].

### 4.6. Oxidation Measurement

#### 4.6.1. Peroxide Value

Peroxide value measurement was performed as described by Richards et al. [69], with some modifications. In each sample, 500 ppm BHT was added to stop the oxidation process and vortexed to achieve homogenisation. Next, 10 mL of CHCl_3_-CH_3_OH (2:1 *v*/*v*) was added to 1 g of the sample. Then, 1.5 mL of NaCl (0.5%) was added, and the samples were vortexed and centrifuged at 2.798× *g* (unicen 21, Ortoalresa, Spain) for 10 min at ambient temperature. The lower phase of CHCl_3_ was collected, and CHCl_3_-CH_3_OH (2:1 *v*/*v*) was added until 10 mL final volume was reached. Following this, 25 μL of NH_4_SCN solution (30% *w*/*v*) and 25 μL of freshly prepared FeCl_2_ solution (0.66% *w*/*v*) were added, and the mixture was vortexed for 2-4 s. A proper amount of the sample was transferred to a Quartz cell, and the absorbance was measured in a spectrometer (uniSPEC 2 UV/VIS-Spectrometer, LLG, Meckenheim, Germany) at 500 nm. Furthermore, 10 mL of CHCl_3_-CH_3_OH (2:1 *v*/*v*) was used as blind. The oxidation products are expressed as mmol/kg of the lipid phase using a standard curve formed with cumene hydroperoxide solutions [70,71,72].

#### 4.6.2. Thiobarbituric Acid Method (TBARS)

The TBARS method was performed according to Lemon [73], with some modifications. First, 1.5 g of the sample was transferred to a test tube containing 5 mL of TCA (7.5% *w*/*v*) and vortexed. The mixture was centrifuged for 30 min at 2.798× *g* (unicen 21, Ortoalresa, Spain). A 2 mL aliquot was mixed with 2 mL of TBA solution (0.02 M). The mixture was heated in a water bath for 40 min at a constant temperature of 100 °C. The samples were then cooled down to room temperature under running tap water and transferred to a Quartz cell to measure the absorbance (uniSPEC 2 UV/VIS-Spectrometer, LLG, Germany) at 532 nm. TBA:TCA solution (1:1 *v*/*v*) was used as blind. The oxidation products are expressed as MDAeq (μmol/L) with the help of the standard curve constructed using TEP solutions. 

### 4.7. Statistical Analysis

Three independent digestion experiments (n = 3) were conducted, and the experimental results are expressed as means ± standard deviations. Minitab 21 Statistical Software (Minitab LLC, State College, PA, USA) was used to statistically process the data by carrying out a one-way ANOVA with Fisher’s test for means comparison. Differences were considered significant at *p* < 0.05.

## Figures and Tables

**Table 1:** Detected vitamin D_3_ content and bioaccessibility indices (BIs) of foods after INFOGEST protocol.

Food Sample		Detected Vitamin D_3_ Content (μg/g ^1^)				Bioaccessibility Index (BI)
		Initial	Thermally Processed	Stomach	Intestine	
Natural	Egg	0.06 ± 0.004 ^b^	0.03 ± 0.005 ^c^	0.08 ± 0.007 ^a^	0.06 ± 0.008 ^b^	1.06 ± 0.153
	Salmon	0.50 ± 0.021 ^c^	0.38 ± 0.020 ^d^	0.74 ± 0.015 ^a^	0.55 ± 0.019 ^b^	1.10 ± 0.060
Fortified	Milk	1.53 ± 0.056 ^a^	N/A	0.62 ± 0.007 ^b^	0.61 ± 0.004 ^b^	0.40 ± 0.015
	Cereals	0.89 ± 0.040 ^a,b^	N/A	0.84 ± 0.005 ^b^	0.92 ± 0.006 ^a^	1.04 ± 0.046
	Sour cherry juice	1.15 ± 0.005 ^c^	N/A	1.20 ± 0.008 ^b^	1.24 ± 0.003 ^a^	1.08 ± 0.054

Table values are means ± standard deviations. Different superscript letters (a, b, c, d) represent statistical differences in the same row (*p* ≤ 0.05). ^1^ g in vitamin D3 content refers to the initial food sample.

**Table 2:** Detected vitamin D_3_ content and bioaccessibility indices (BIs) at four different gastric pH values after INFOGEST application.

Gastric pH Value	Detected Vitamin D_3_ Content (μg/mL)			BI
	Initial	Stomach	Intestine	
1	95.93 ± 0.64 ^a^	39.87 ± 8.97 ^b,B^	70.86 ± 4.58 ^c,A^	0.74 ± 0.05
3		40.95 ± 2.69 ^b,B^	51.71 ± 5.46 ^c,B^	0.54 ± 0.06
5		47.14 ± 3.71 ^b,A,B^	51.62 ± 2.08 ^b,B^	0.54 ± 0.02
7		53.65 ± 6.55 ^b,A^	41.28 ± 2.89 ^c,C^	0.43 ± 0.03

Table values are means ± standard deviations. Different superscript lowercase letters (a, b, c) represent statistical differences (*p* ≤ 0.05) in the same row. Different superscript uppercase (A, B, C) letters represent statistical differences (*p* ≤ 0.05) in the same column.

## Data Availability

Data are available upon request to the corresponding author: pkvareltzis@cheng.auth.gr.

## Supplementary Materials

The following supporting information can be downloaded at: https://www.mdpi.com/article/10.3390/molecules29051153/s1, Table S1. Average peak areas (n = 3) of low concentrations of vitamin D_3_ standard; Table S2. LINEST function parameters; Figure S1: Vitamin D_3_ standard curve; Figure S2: Vitamin D_3_ standards for standard curve; Figure S3: Raw salmon sample spiked with vitamin D_3_; Figure S4: Raw egg sample spiked with vitamin D_3_; Figure S5: Chromatograph of liquid supplement after gastric digestion at pH 1. (A), (B), and (C) may be isomers of vitamin D_3_, produced during digestion due to acidic degradation.
