# Supplementary material for: Vitamin D3 Bioaccessibility from Supplements and Foods—Gastric pH Effect Using a Static In Vitro Gastrointestinal Model
Source: Molecules. 2024 Mar 5;29(5):1153. doi: 10.3390/molecules29051153 (PMC10934191; doi:10.3390/molecules29051153)
Supplement: Supplementary file 1 [file molecules-29-01153-s001.zip › molecules-2871719-supplementary.pdf]

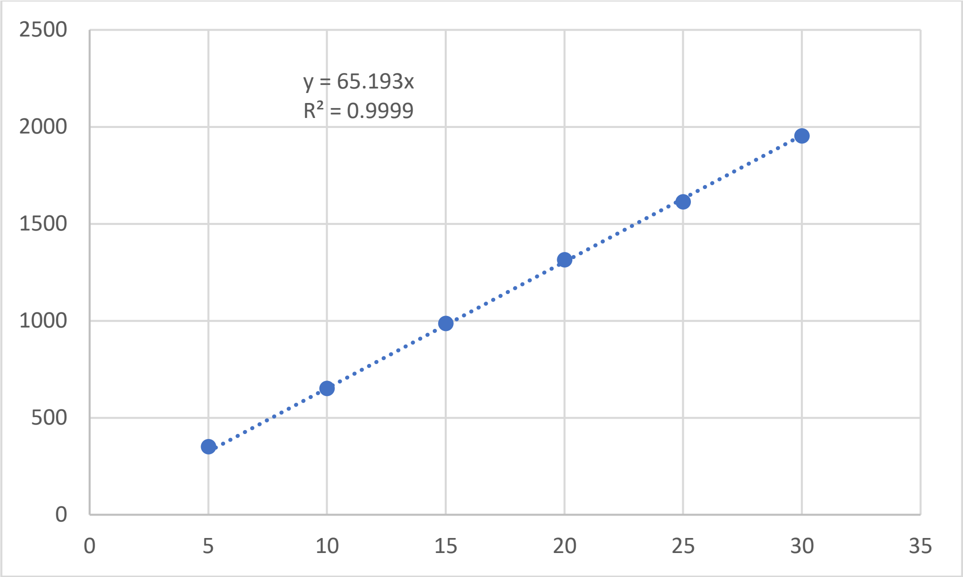

Figure S1. Vitamin D_3 standard curve

Table S1. Average peak areas (n=3) of low concentrations of vitamin D_3 standard

Concentration (ppm)	Response
0.05	2.75
0.1	6.4
0.15	6.91
0.2	10.98
0.25	14.28
0.3	15.83

Table S2. LINEST function parameters

Parameter	Value
Slope of standard curve	53.21
Standard deviation of response	0.88

The limit of detection (LOD) and limit of quantification (LOQ) are calculated based on the following equations:

$$LOD = 3.3 * S_y/S$$

$$LOQ = 10 * S_y/S$$

Where S_y is the standard deviation of the response and S is the slope of the standard curve.

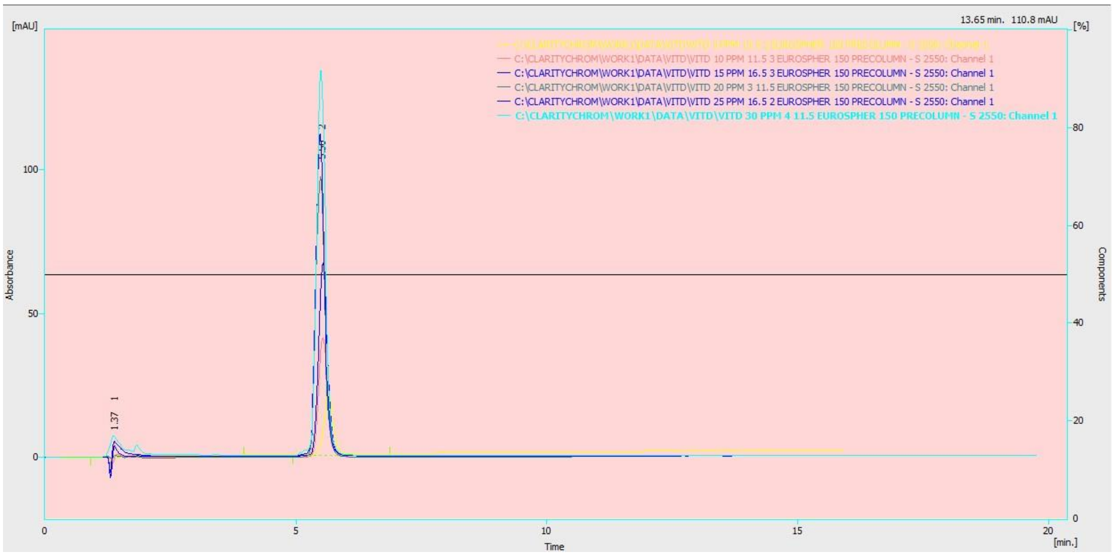

Figure S2. Vitamin D_3 standards for standard curve

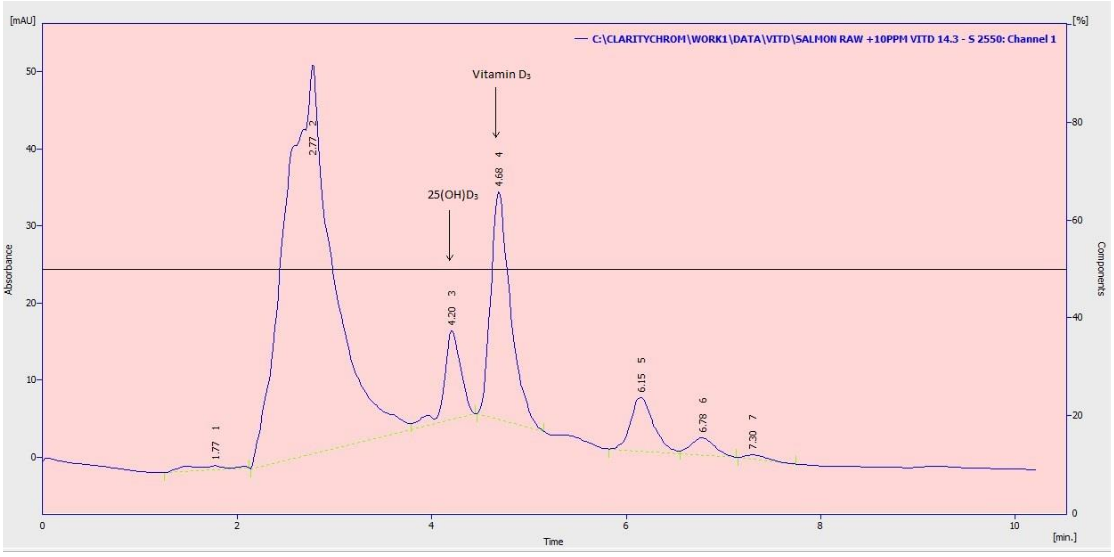

Figure S3. Raw salmon sample spiked with vitamin D_3.

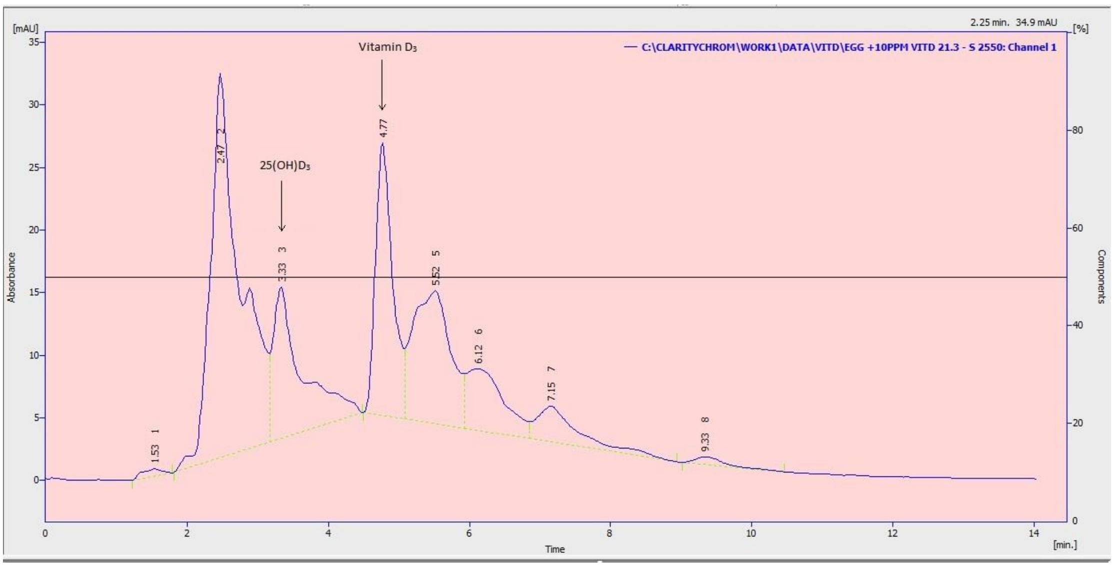

Figure S4. Raw egg sample spiked with vitamin D_3.

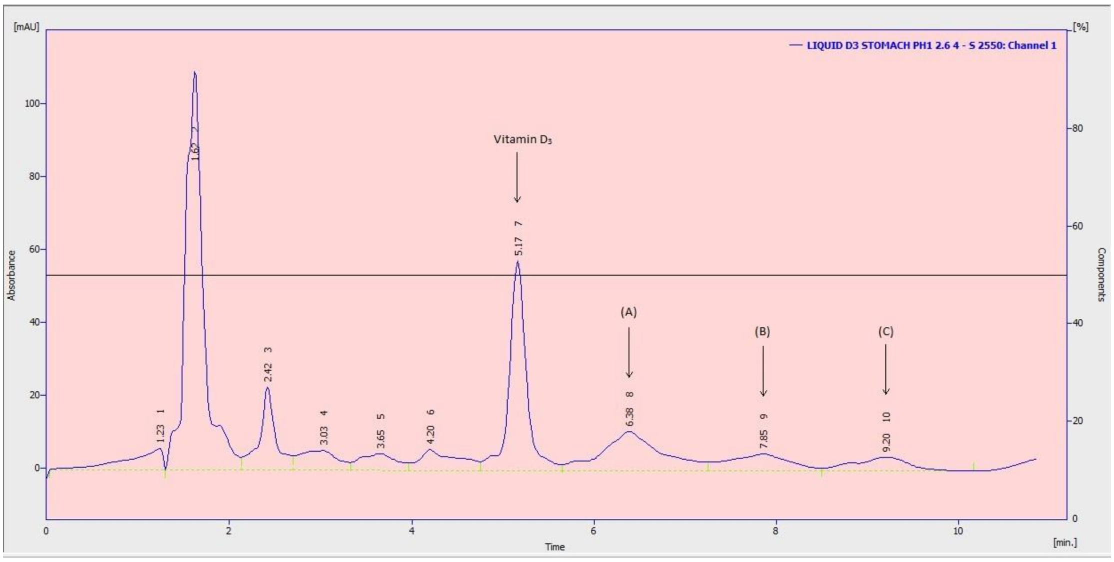

Figure S5. Chromatograph of liquid supplement after gastric digestion at pH 1. (A), (B) and (C) may be isomers of vitamin D_3, produced during digestion due to acidic degradation.
